# Antioxidant Activity Derived from Marine Green-Lipped Mussel *Perna canaliculus* Extracts in Mice

**DOI:** 10.1155/2021/1622270

**Published:** 2021-08-06

**Authors:** Ahmad A. Alghamdi, Ayman Al-Hazmi, Abdulraheem A. Almalki, Asma A. Alsubaihi, Sulaiman A. Anagreyyah, Ahmed H. Qasem, Nuha A. Anajirih, Mamdouh Allahyani, Reema A. Alyamani, Mohammad A. Albanghali, Yasser M. Kofiah, Haitham A. Bukhary, Abdullah F. Aldairi

**Affiliations:** ^1^Department of Clinical Laboratories Sciences, College of Applied Medical Sciences, Taif University, P.O. Box 11099, Taif 21944, Saudi Arabia; ^2^Faculty of Medicine, Biochemistry Department, Umm Al-Qura University, Al Abdeyah, PO Box 7607, Makkah, Saudi Arabia; ^3^Preventive Medicine Department, King Fahad Armed Force Hospital, Al Andalus, P.O. Box 23311, Jeddah, Saudi Arabia; ^4^Laboratory Medicine Department, Faculty of Applied Medical Sciences, Umm Al-Qura University, Al Abdeyah, PO Box 7607, Makkah, Saudi Arabia; ^5^Medical Emergency Services Department, Faculty of Health Sciences, Umm Al-Qura University, P.O. Box 1109, Al-Qunfudah, Saudi Arabia; ^6^Clinical Nutrition Department, Faculty of Applied Medical Sciences, Umm Al-Qura University, Al Abdeyah, PO Box 7607, Makkah, Saudi Arabia; ^7^Department of Public Health, Faculty of Applied Medical Sciences, Albaha University, Albaha, Saudi Arabia; ^8^Department of General Surgery, Faculty of Medicine, Albaha University, Albaha, Saudi Arabia; ^9^Department of Pharmaceutics, College of Pharmacy, Umm Al-Qura University, Makkah 24381, Saudi Arabia

## Abstract

This study investigates the antioxidant activities of lipid, protein, and carbohydrate extracts from the marine mollusk *Perna canaliculus*. Lipids were extracted using acetone, which was followed by protein extraction using the broad-spectrum enzyme Alcalase and then carbohydrate extraction using cetylpyridinium chloride. Eighty white BALB/c mice were divided into eight groups according to the administered extracts. Groups 1 and 5 were the control and toxin control groups, respectively. Groups 2, 3, and 4 were administered lipid, protein, and carbohydrate extracts, respectively. The other groups were administered *P. canaliculus* extracts as well as gentamicin and acetaminophen, known as ethanolic extracts, derived from Nerium oleander to induce oxidation stress. All groups showed significant improvements in body weight (*p* < 0.05). The lipid extract group showed a significant decrease in low-density lipoprotein cholesterol (*p* < 0.05) and a significant increase in high-density lipoprotein cholesterol (*p* < 0.05). After the toxin injection, all groups treated with *P. canaliculus* extracts showed increased antioxidant effects on hepatocytes (*p* < 0.05). The lipid extracts induced antioxidant effects to protect the kidney by increasing lipid peroxidation (*p* < 0.05) and catalase activities (*p* < 0.05). Also, protein extracts showed antioxidant effects by increasing glutathione and catalase levels significantly (*p* < 0.005). In conclusion, *P. canaliculus* extracts, especially lipids and proteins, have potent antioxidant activities that protect vital organs from oxidation stress.

## 1. Introduction

Oxygen's interaction with cellular molecules produces highly reactive elements known as reactive radicals, which can make oxidative modifications to cells' macromolecules that would lead to cell injury [1]. There are two sources of reactive oxygen species (ROS): endogenous and exogenous. For instance, a well-known endogenous ROS is nicotinamide adenine dinucleotide phosphate (NADPH) oxidase, which is formed during cellular respiration. The NADPH oxidase is known as the dominant source of the superoxide anion (O_2_^·^), which could be disproportionate via superoxide dismutase (SOD) to produce hydrogen peroxide (H_2_O_2_); thus, it could become a reactive hydroxyl group (OH^-·^) via the Haber-Weiss reaction. Therefore, it would be extremely reactive as it attaches to cellular macromolecules, resulting in cellular death. The exogenous sources of ROS can be accumulated through tobacco smoking and alcohol consumption, among others [2]. However, the human body has counter mechanisms against oxidative stress caused by ROS via antioxidants. Antioxidants are substances with a crucial role in protecting cellular organelles against oxidation stress, which can progress during inflammation or other disorders [3]. There are two types of antioxidants: endogenous (obtained through the body's metabolism) and exogenous (derived from one's diet). Endogenous antioxidant activity occurs via two pathways: enzymatic and nonenzymatic. For instance, the formation of the highly reactive hydroxyl group produced via SOD from H_2_O_2_ can be prevented when the catalase enzyme, an endogenous antioxidant, breaks down H_2_O_2_ into oxygen and water. Moreover, when reduced glutathione (GSH) is oxidized to glutathione disulfide, the antioxidant agent glutathione peroxidase (GSH-Px) will reduce oxidized glutathione and, thus, help protect cells from oxidative stress [2]. Several antioxidant agents obtained from nature, such as plants and marine life, promote antioxidant activities to diminish the effects of oxidative stress on human cells [4, 5].

Biologically active compounds from marine life were widely discussed in the literature due to their potential anticancer [4, 6–9], antiviral [10], and antithrombosis [7, 11] activities. This study evaluated the role of extracts from *Perna canaliculus* (green-lipped mussel) as potential antioxidant agents. Several studies examined mussels and their antioxidant materials [12, 13], as well as the effect of oxidation stress on the mussels themselves [14, 15]. Therefore, in this study, we aimed to investigate the antioxidant effects of *P. canaliculus* extracts on induced mouse toxicity.

## 2. Materials and Methods

### 2.1. Chemicals

All chemicals were purchased from Sigma-Aldrich, USA, unless otherwise stated.

### 2.2. Polar-Lipid Extracts

Lipids were extracted from *P. canaliculus*, obtained from New Zealand, immediately frozen, and shipped to Saudi Arabia via SeaLand Company®. Initially, the whole body was thawed at room temperature. The shells were removed, and 4 kg of the whole soft body tissue was oven-dried at 70°C under vacuum for 8 hours (Sheldon®, Inc.). Dried tissues were blended into a fine powder using a razor blade. Then, 1 kg of powder was submerged in acetone for 72 hours, and acetone was changed every 24 hours, ensuring full-fat removal [6, 16]. Acetone was collected and dried under a vacuum. Afterward, lipid extract was stored at -20°C in a sterile container for further analysis.

### 2.3. Protein Extracts

Protein extraction was performed on the lipid-free *P. canaliculus* powder, followed by acetone extraction. Using wide-spectrum Alcalase® enzyme (Merck Millipore, Watford, UK), 50 g of lipid-free mussel powder was incubated for 48 hours at 60°C. Then, protein residues were precipitated using 5% trichloroacetic acid (TCA) [6], and the supernatant was used to extract sulfated polysaccharides. Afterward, 400 *μ*L of the radioimmunoprecipitation assay (RIPA) buffer (Thermo Scientific, USA) was added to about 4 g of the precipitated protein, and the mixture was vortexed. Then, the sample was stored in the fridge for 15 minutes. Finally, the protein sample was centrifuged at 15,000 rpm for 45 minutes at 4°C. The protein supernatant was stored at -20°C for further analysis.

### 2.4. Sulfated Polysaccharide Extracts

Following the protein extraction, after the addition of 5% TCA, the supernatant was following the protocol from [6], which used cetylpyridinium chloride as a part of the standard procedure to extract sulfated glycans [16].

### 2.5. Preparation of the *Nerium oleander* Extract

The *Nerium oleander* tree was purchased from a local plant shop. Both the pink flowers and green leaves were washed with water and then dried at 40°C using a drying oven (Sheldon®, Inc.). The dried flowers and leaves were ground into a fine powder; 500 g of the powder was dissolved in 90% ethanol for 7 days and filtered several times using Whatman® filter paper. Eventually, the extract was stored in a glass container at 5°C.

### 2.6. Animals

The experiment was conducted in accordance with the EU Directive 2010/63/EU recommendations for animal research after the approval of the Biomedical Research Ethics Committee at Umm Al-Qura University's Faculty of Medicine (ethical approval number: HAPO-02-K-012-2021-03-614). The white BALB/c mice with an average weight of 19–21 g and ages between 6 and 8 weeks were obtained from a local rodent market. The mice were housed in polycarbonate cages (10 mice/cage) on a woodchip bedding. They were maintained in a large and ventilated room with an automatic 12-hour light/dark cycle and at a temperature of 23 ± 2°C. These mice had free access to water and were fed ordinary rodent chow during the two weeks of acclimatization.

### 2.7. Experimental Design

Following the two weeks of acclimatization, 80 mice were randomly classified into eight groups (10 mice/group) as follows:

- Group 1 (control group): ten mice received normal rodent chow and ordinary bottled water
- Group 2 (*P. canaliculus* lipid extracts): ten mice were fed normal rodent chow and drank ordinary bottled water. The mice were administered *P. canaliculus* lipid extracts 500 mg/kg body weight through intragastric gavage
- Group 3 (*P. canaliculus* protein extracts): ten mice were fed normal rodent chow and drank ordinary bottled water. They were *administered P. canaliculus* protein extracts 500 mg/kg body weight through intragastric gavage
- Group 4 (*P. canaliculus* sulfated polysaccharide extracts): ten mice were fed normal rodent chow and drank ordinary bottled water. They were administered *P. canaliculus* sulfated polysaccharide extract 500 mg/kg body weight through intragastric gavage
- Group 5 (toxin control group): ten mice were fed a normal rodent diet and drank ordinary bottled water. Each mouse was administered N. oleander ethanolic extract through intragastric gavage at a dosage of 200 mg/kg body weight twice a week. At the end of the experiment, the mice were injected intraperitoneally with a mixture of acetaminophen (Panadol baby drops 100 mg/mL) 300 mg/kg body weight and gentamicin (baby drops) 50 mg/kg body weight after overnight fasting (12 hours) twice a week
- Group 6 (toxin and *P. canaliculus* lipid extracts): ten mice had a normal rodent diet and drank bottled water. These mice were administered *P. canaliculus* lipid extracts in the same way as group 2 and N. oleander ethanolic extracts. At the end of the experiment, the mice were injected with a mixture of acetaminophen and gentamicin, like in group 5
- Group 7 (toxin and *P. canaliculus* protein extracts): ten mice had a normal rodent diet and drank bottled water. These mice were administered *P. canaliculus* protein extracts in the same way as group 3 and N. oleander ethanolic extracts. At the end of the experiment, the mice were injected with a mixture of acetaminophen and gentamicin, like in group 5
- Group 8 (toxin and *P. canaliculus* sulfated polysaccharide extracts): ten mice had a normal rodent diet and drank bottled water. These mice were administered *P. canaliculus* sulfated polysaccharide extracts in the same way as group 4 and N. oleander ethanolic extracts. At the end of the experiment, the mice were injected with a mixture of acetaminophen and gentamicin, like in group 5

### 2.8. Biochemical Analysis

On day 61, a blood sample was collected from each rat from their portal vein in a plain tube (no preservatives). The samples were allowed to coagulate for 30 minutes and were centrifuged at 2,500 rpm for 15 minutes. Then, serum samples were collected. Using HumaStar® (HUMAN, Germany), a panel of biochemistry analysis was performed for each sample, including glucose, urea, creatinine, aspartate aminotransferase (AST), alanine aminotransferase (ALT), alkaline phosphatase (ALP), cholesterol (Chol), high-density lipoprotein cholesterol (HDL-C), low-density lipoprotein cholesterol (LDL-C), creatine kinase (CK), creatine kinase-myocardial band (CK-MB), and troponin-I.

### 2.9. Hematological Analysis

On day 61, blood samples were collected from mice's portal veins in ethylenediaminetetraacetic acid (EDTA) tubes. A panel of hematological analysis was performed for each sample using a complete blood count (CBC) Mindray BC-2800 analyzer. This analysis includes testing for the red blood cell (RBC) count, the hemoglobin (Hb) count, the hematocrit level, and the mean cell volume (MCV).

### 2.10. Preparation of Tissue Homogenate

Liver and kidney homogenates were prepared using a 2 mL bead Ruptor kit microtube (Omni International, USA). The RIPA buffer with a protease inhibitor and a piece of the organ was added into the microtube, which was placed on a homogenate machine (Omni International, USA) for 2 minutes. Then, the microtube was centrifuged at 8°C at 15,000 rpm for 30 minutes (Sigma-Aldrich, USA). The supernatant was aspirated into a microtube and centrifuged at 8°C at 15,000 rpm for 15 minutes. It was then aspirated and stored at -20°C for further analysis.

### 2.11. Estimation of Tissue Homogenate Antioxidants and Oxidant Parameters

#### 2.11.1. Lipid Peroxide (LPO) Estimation

Lipid peroxidation was estimated through the reaction of thiobarbituric acid with malondialdehyde (Elabscience, USA) in an acidic medium at 95°C for 30 minutes. The product called thiobarbituric acid was detected at 534 nm using Varioskan™ LUX (Thermo Fisher Scientific, USA).

#### 2.11.2. Reduced Glutathione Estimation

Reduced glutathione in the sample was reacted with dinitrobenzoic acid (Elabscience, USA) to form a yellow complex, which was detected at 405 nm using Varioskan™ LUX (Thermo Fisher Scientific, USA). GSH at 1 *μ*mol/L was consumed by 1 mg of GSH-Px (Elabscience, USA) at 37°C for 5 minutes, forming a coloring product that was detected at 412 nm using Varioskan™ LUX (Thermo Fisher Scientific, USA).

#### 2.11.3. Glutathione Peroxidase (GSH-Px) Estimation

1 *μ*mol/L of GSH was consumed by 1 mg of GSH-Px (Elabscience, USA) at 37°C for 5 minutes, forming a coloring product that was detected at 412 nm using Varioskan™ LUX (Thermo Fisher Scientific, USA).

#### 2.11.4. Estimation of Superoxide Dismutase (SOD)

The SOD activity was estimated using the water-soluble tetrazolium-1 (WST-1) method (Elabscience, USA). The reaction of WST-1 with oxygen to generate a water-soluble formazan was catalyzed by xanthine oxidase. This reaction was inhibited by SOD in the sample, so the SOD activity was inversely correlated with the amount of formazan dye using Varioskan™ LUX (Thermo Fisher Scientific, USA). The reaction inhibition ratio by SOD was calculated as the following: *i* = ([*A*] control − [*A*] blank)–([*A*] sample − [*A*] blank) ÷ ([*A*] control − [*A*] blank) × 100, where *i* stands for inhibition and *A* stands for absorbance. SOD activity then was calculated as the following: T‐SOD activity (*μ*g/g protein) = *I* ÷ 50% × (V1 ÷ V2) × *f* ÷ concentration of the protein, where V1 is the total volume of the reaction, V2 is the volume of sample added to the reaction, and *f* is the dilution factor.

### 2.12. Statistical Analysis

All data analysis was done using GraphPad Prism version 8.2.1 (San Diego, CA, USA). One-way ANOVA analyzed data to compare hematological and biochemical parameters among different groups, as the significance level was set at *p* < 0.05. The antioxidant data were analyzed using Statistical Package for the Social Sciences (SPSS) version 16 (SPSS Inc., Chicago, IL, USA). All data are presented as the mean ± standard deviation (SD).

## 3. Results and Discussion

The mice in each group were weighed in the experiment (Table 1). The lipid, protein, and carbohydrate extract groups showed a significant statistical difference in body weight compared with the control group after four weeks and at the end of the experiment (*p* < 0.05). Both the lipid and carbohydrate extract groups showed a higher increase in body weight than the protein extract group. The CBC did not show significant statistical differences between all groups, which may be due to the short experiment duration (Table 2).

According to the biochemical tests, the cholesterol and LDL-C levels were significantly reduced in the lipid fraction group compared to the control group, while the HDL-C level was significantly higher in the same group (*p* < 0.05) (Table 3). In the liver function tests, the lipid, protein, and carbohydrate fraction groups had significantly reduced serum ALT, AST, and ALP activities after the toxin administration compared with the toxin group (*p* < 0.05). The lowest ALT and AST activities were observed in the lipid fraction group, while the lowest ALP activity was associated with the protein fraction group. Moreover, the lipid, protein, and carbohydrate fraction groups showed significantly reduced total CK, CK-MB, and c-troponin I after the toxin administration compared with the toxin group (*p* < 0.05). The lower total CK and CK-MB activities were associated more with the lipid extract group than with the toxin group (*p* < 0.05). Furthermore, the lipid fraction group had significantly lower c-troponin levels after the toxin administration than the toxin group (*p* < 0.05).

Regarding the oxidative products and antioxidants in the hepatic homogenate, lipid peroxidation levels were significantly lower in the *P. canaliculus* extract group than in the toxin group (*p* < 0.05). All three extract groups significantly increased the reduced glutathione levels compared with the toxin group (*p* < 0.05). The highest level was present in mice treated with protein extracts. In addition, the catalase activity was more significantly induced in lipid, protein, and carbohydrate extract groups administered with toxins than in the toxin group, with the highest level observed in the protein fraction group (*p* < 0.01). Moreover, the GSH-Px activity was significantly induced in mice injected with toxins and treated with protein extracts (*p* < 0.05) (Table 4).

In the kidney homogenate, lipid peroxidation was significantly lower in the lipid extract group than in the toxin group (*p* < 0.05). The reduced glutathione level and catalase activity were more significantly induced in mice treated with lipid extracts than in mice injected with toxins (*p* < 0.05 and *p* < 0.01, respectively) (Table 5). In the cardiac homogenate, lipid peroxidation was significantly lower in mice injected with toxins and treated with lipid extracts (*p* < 0.05), while the reduced glutathione level and catalase activity were significantly induced in the protein extract group than in the toxin group (*p* < 0.01) (Table 6).

*N. oleander* is a known cardiotoxin due to its glycoside content [17]. The present study used a mixture of toxins, acetaminophen, gentamicin, and *N. oleander* ethanolic extracts to induce oxidative stress. The lipid, carbohydrate, and protein extracts from *P. canaliculus* induced body weight gain in mice. However, the carbohydrate fraction group had the highest body weight without changing the blood glucose level. This agrees with the phenomenon that the consumption of a carbohydrate diet results in body weight gain more than the consumption of the same amount of lipid and protein separately, as carbohydrates consumed in excess will be stored in adipose tissue. In the present study, the consumption of carbohydrate, lipid, and protein extracts of *P. canaliculus* by different groups did not affect each mouse's hematological parameters, which could be due to the short duration of the experiment. The lipid extracts of the marine mollusk increased HDL-C and reduced LDL-C in the mice. Therefore, this fraction of *P. canaliculus* is beneficial in reducing risks for coronary heart disease and atherosclerosis. It might be due to *P. canaliculus*' high content of polyunsaturated fatty acids, mainly omega-3 fatty acids.

The lipid-rich extract of *P. canaliculus* has five lipid classes: free fatty acids, steroids, triglycerides, sterol esters, and polar lipids. Free fatty acids include saturated fatty acids and omega-3 polyunsaturated fatty acids [3, 5]. Polyunsaturated fatty acids show anti-inflammatory action through the degradation of phospholipases and lipoxygenases [14, 15]. Moreover, these fatty acids induce an anti-inflammatory process through improved antioxidant activity [1, 12].

**Figure 1:**
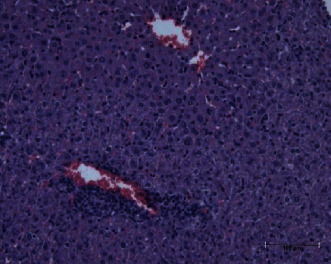
Toxin group liver showed polymorphs and mononuclear inflammatory cell infiltration around the central vein. Hepatocyte appears with cytoplasmic vacuoles (fat droplets) and a condense nucleus with some necrotic cells.

**Figure 2:**
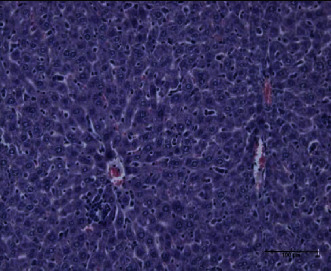
Carbohydrates and toxin group liver showed congested hepatic artery and portal vein. The portal tract is infiltrated with lymphocytes. The sinusoids showed slight hemorrhage.

**Figure 3:**
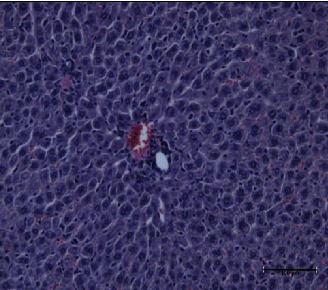
Lipid and toxin group liver showed normal central venule surrounded by normal hepatocyte with normal nucleus. The sinusoids showed proliferated Kupffer's cells.

**Figure 4:**
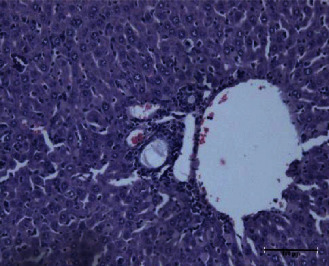
Proteins and toxin group liver showed normal hepatocytes with a normal nucleus. The central vein, hepatic artery, and portal vein are congested and dilated. The sinusoids showed Kupffer's cell proliferation.

The present study showed that the administration of protein extracts reduced ALT and AST activities after injecting mice with toxins. The lipid fraction reduced the LPO level in the liver homogenate after the toxin injection, the glutathione level, and the activities of catalase and GSH-Px. The livers of mice in the toxin group showed inflammatory cell infiltration at three zones around the central vein (Figure 1). The hepatocytes were vacuolated with the abnormal condensed nucleus. There was an area of cellular necrosis, which was also present in the liver of mice treated with carbohydrate extracts after toxin injection (Figure 2). The livers of mouse groups injected with toxins, and then *P. canaliculus* lipid (Figure 3), and protein extracts (Figure 4) did not show any pathologic appearance. The hepatocytes normally appeared with normal nuclei and without vacuoles or evidence of necrosis. Furthermore, there was no inflammatory cell infiltration either in the portal tract or in any zone around the central vein.

A previous study showed that the active peptides extracted by the pepsin digestion of *P. canaliculus* worked as a radical scavenger molecule [18]. In the present study, the protein extract group showed reduced ALP activity after the toxin injection compared with the toxin group. The liver of the mice in this group showed normal hepatocytes without any evidence of necrosis and normal sinusoids. There was a congested central vein, portal vein, and hepatic artery (Figure 4).

**Figure 5:**
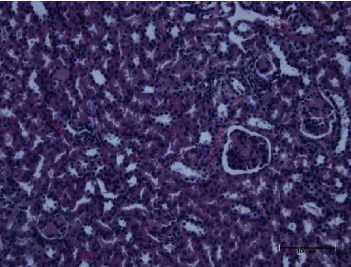
Toxin group kidney showed marked congested dilated blood vessel and hyaline materials in glomeruli.

**Figure 6:**
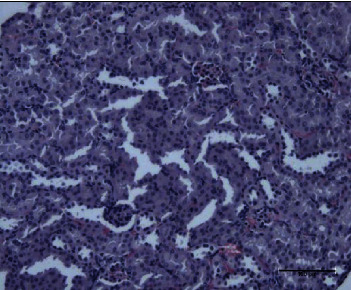
Lipid and toxin group kidney appears with normal glomeruli with normal tuft and mesangial cells. The tubules appear normally with normal interstitial tissue.

**Figure 7:**
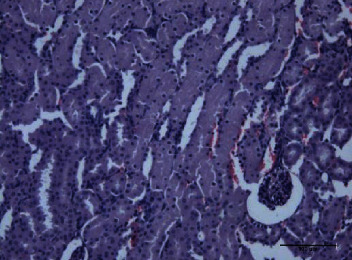
Proteins and toxin group kidney showed normal glomeruli appearance with normal tubules. There are some areas of hemorrhage within interstitial tissue.

**Figure 8:**
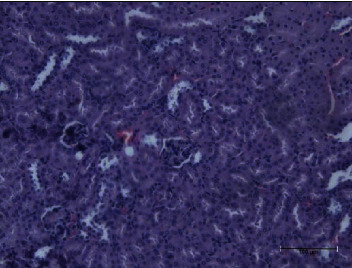
Carbohydrates and toxin group kidney showed dilated and congested blood vessels with some hemorrhage area. The glomeruli appear normally without any abnormal changes. The tubules showed hyaline material. The interstitial tissue shows some hemorrhage area without any inflammatory cell infiltration.

In the kidney homogenate, the lipid fraction of *P. canaliculus* significantly reduced the LPO level after the toxin injection. Both lipid and protein fractions induced reduced glutathione level and catalase activity in different groups. In the histological examination of the kidney, the toxin group showed congested blood vessels with hyaline casts (Figure 5); however, both lipid (Figure 6) and protein (Figure 7) groups after the injection of toxins showed normal glomeruli with normal tubules and without hyaline material aggregation. There was only a slight hemorrhage in the interstitial tissue of the protein fraction group (Figure 7) and congested blood vessels with hemorrhage in the carbohydrate group (Figure 8).

**Figure 9:**
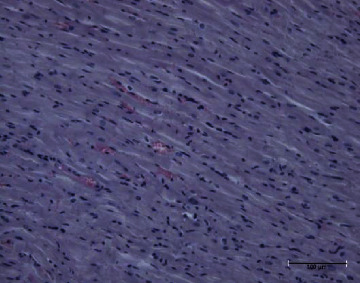
Lipid and toxin group heart showed normal cardiomyocytes with normal nuclei. There are normal cardiac fibers with normal striation.

**Figure 10:**
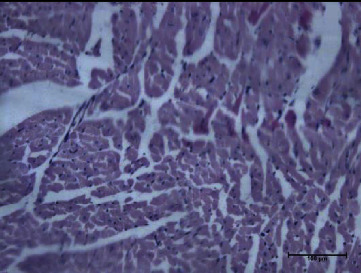
Carbohydrates and toxin group heart showed cardiomyocyte necrosis and loss of normal striation. Moreover, there is dilated blood vessel.

**Figure 11:**
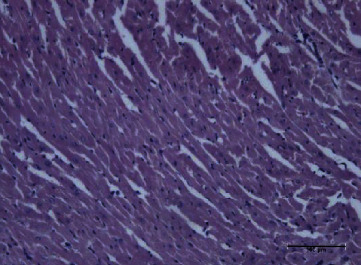
Proteins and toxin group heart showed normal cardiomyocytes with normal nuclei. There are normal cardiac fibers with normal striation without any area of hemorrhage.

**Figure 12:**
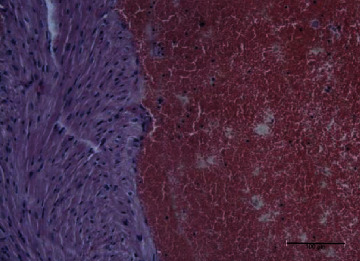
Toxin group heart showed massive hemorrhage with cardiomyocyte necrosis and loss of normal striation.

In the cardiac homogenate, the lipid extracts of *P. canaliculus* reduced the LPO level after the toxin injection, while the protein extracts induced reduced glutathione level and catalase activity. The lipid fraction group also showed reduced CK and CK-MB activities and troponin I level after the injection of toxins. The hearts of the mice in this group showed normal cardiomyocytes with normal nuclei, as well as normal cardiac fibers with normal striation (Figures 9 and 10). The hearts of those in the protein fraction group after toxin injection showed normal cardiomyocytes with normal nuclei, plus normal cardiac fibers with normal striation and without any hemorrhage (Figure 11), compared with the toxin group which shows massive hemorrhage (Figure 12).

## 4. Conclusion

The lipid and protein fractions of the *P. canaliculus* protect the liver, heart, and kidney from different types of toxins by their antioxidant activities. Moreover, the lipid fraction of marine may have antiatherosclerotic activity by lowering LDL-C and induction of HDL-C.

## Figures and Tables

**Table 1:** Changes in body weight of mice in each group during the experiment.

	Control group	Toxin group	Lipid group	Lipid+toxin group	CHO group	CHO+toxin group	Protein group	Protein+toxin group	*p* value
In the beginning of the experiment	19.96 ± 1.37	19.87 ± 1.22	20.15 ± 2.38	19.33 ± 1.30	21.49 ± 1.29	19.70 ± 1.11	19.98 ± 1.39	19.66 ± 1.19	0.099
After four weeks	21.82 ± 1.69	19.06 ± 1.03	27.23 ± 2.79	22.08 ± 1.44	28.64 ± 2.07	20.06 ± 1.53	24.05 ± 2.02	20.18 ± 1.50	0.042^∗^
At the end of the experiment	24.56 ± 1.81	16.01 ± 1.66	28.18 ± 2.13	22.51 ± 1.07	30.86 ± 3.04	21.41 ± 1.41	27.33 ± 2.12	21.22 ± 1.06	0.031^∗^

^∗^*p* < 0.05; ^∗∗^*p* < 0.01.

**Table 2:** Hematological parameters for all groups.

	Control group	Toxin group	Lipid group	Lipid+toxin group	CHO group	CHO+toxin group	Protein group	Protein+toxin group	*p* value
RBCs (10^6^) (*μ*L)	7.34 ± 0.51	7.88 ± 0.60	7.76 ± 0.61	7.62 ± 0.42	7.91 ± 0.64	7.81 ± 0.71	7.71 ± 0.77	7.21 ± 0.33	0.080
Hemoglobin (g/dL)	10.28 ± 0.56	10.18 ± 0.55	10.23 ± 0.35	10.33 ± 0.41	10.29 ± 0.71	10.11 ± 0.49	10.55 ± 0.38	10.31 ± 0.40	0.085
Hematocrit (%)	41.77 ± 0.59	38.66 ± 0.92	41.56 ± 0.99	3972 ± 0.48	41.16 ± 0.83	39.47 ± 0.60	41.25 ± 0.51	38.91 ± 0.58	0.087
MCV (fL)	50.28 ± 0.72	48.50 ± 0.78	50.36 ± 0.65	50.66 ± 0.93	50.09 ± 0.44	49.50 ± 0.81	50.29 ± 0.90	48.99 ± 0.82	0.091

^∗^*p* < 0.05; ^∗∗^*p* < 0.01.

**Table 3:** Biochemical parameters: serum glucose, total cholesterol, HDL-C, LDL-C, ALT, AST, ALP, urea, creatinine, total CK, CK-MB, and cardiac troponin-I for mice in all groups.

	Control group	Toxin group	Lipid group	Lipid+toxin group	CHO group	CHO+toxin group	Protein group	Protein+toxin group	*p* value
Glucose (mmol/L)	4.30 ± 0.32	4.80 ± 0.57	4.51 ± 0.48	4.07 ± 0.93	4.84 ± 1.81	4.18 ± 0.37	4.80 ± 0.91	4.10 ± 0.42	0.081
Cholesterol (mmol/L)	2.02 ± 0.45	2.82 ± 0.22	1.61 ± 0.36	2.20 ± 0.87	2.50 ± 0.61	2.30 ± 0.47	2.00 ± 0.82	2.40 ± 0.58	0.041^∗^
HDL-C (mmol/L)	1.54 ± 0.62	1.02 ± 0.44	2.02 ± 0.33	1.81 ± 0.51	1.66 ± 0.41	1.50 ± 0.52	1.93 ± 0.40	1.50 ± 0.37	0.038^∗^
LDL-C (mmol/L)	2.56 ± 0.70	2.90 ± 0.63	0.20 ± 0.03	2.30 ± 0.32	1.87 ± 0.21	1.96 ± 0.41	0.30 ± 0.07	0.95 ± 0.11	0.033^∗^
ALT (U/L)	66.71 ± 6.28	234.60 ± 28.61	60.22 ± 7.65	83.27 ± 17.71	74.60 ± 18.01	121.39 ± 18.90	61.60 ± 11.61	124.27 ± 14.71	0.044^∗^
AST (U/L)	81.41 ± 17.22	751.45 ± 34.90	62.77 ± 19.60	127.81 ± 34.84	83.45 ± 20.11	219.81 ± 41.07	56.22 ± 13.90	244.81 ± 25.84	0.028^∗^
ALP (U/L)	98.64 ± 18.01	258.33 ± 58.16	94.28 ± 21.22	82.33 ± 21.20	122.33 ± 29.22	141.33 ± 30.52	70.31 ± 16.14	112.33 ± 21.20	0.039^∗^
Urea (mg/dL)	22.57 ± 2.78	61.11 ± 11.96	20.11 ± 3.91	44..02 ± 8.39	25.73 ± 9.22	48..82 ± 9.66	18.91 ± 3.91	36.22 ± 5.31	0.052
Creatinine (mg/dL)	0.66 ± 0.26	0.965 ± 0.77	0.610 ± 0.04	0.996 ± 0.41	0.710 ± 0.11	0.771 ± 0.21	0.53 ± 0.26	0.803 ± 0.21	0.070
CK (U/L)	191.22 ± 15.33	423.67 ± 52.76	180.18 ± 22.55	226.88 ± 33.71	201.31 ± 32.06	311.80 ± 40.12	182.33 ± 21.52	303.88 ± 33.71	0.029^∗^
CK-MB (U/L)	39.77 ± 2.17	165.37 ± 20.51	34.60 ± 3.87	72.62 ± 10.67	41.22 ± 9.42	102.27 ± 18.04	32.11 ± 5.87	109.62 ± 10.67	0.045^∗^
c-troponin I	6.71 ± 0.72	31.90 ± 7.11	5.08 ± 1.06	8.22 ± 5.44	6.01 ± 0.62	23.04 ± 5.44	15.02 ± 0.64	18.90 ± 3.44	0.040^∗^

^∗^*p* < 0.05; ^∗∗^*p* < 0.01. ALT: alanine aminotransferase; AST: aspartate aminotransferase; ALP: alkaline phosphatase; CK: creatinine kinase; CK-MB: creatinine kinase-MB.

**Table 4:** Estimation of tissue homogenate lipid peroxidation (LPO), reduced glutathione (GSH), catalase, glutathione peroxidase (GSH-Px), and superoxide dismutase (SOD) in liver tissue for all groups.

	Control group	Toxin group	Lipid group	Lipid+toxin group	CHO group	CHO+toxin group	Protein group	Protein+toxin group	*p* value
LPO (nmol/g)	22.53 ± 4.64	56.76 ± 11.52	11.27 ± 2.09	36.57 ± 5.33	15.54 ± 3.61	30.91 ± 5.11	13.66 ± 2.26	29.97 ± 6.20	0.028^∗^
GSH (nmol/g)	4.42 ± 0.34	1.88 ± 0.52	3.44 ± 0.39	5.70 ± 0.41	3.09 ± 0.28	3.33 ± 0.21	3.32 ± 0.24	7.68 ± 0.38	0.040^∗^
Catalase (U/g)	77.21 ± 14.11	28.53 ± 7.04	102.77 ± 20.07	80.30 ± 6.11	95.55 ± 31.04	66.02 ± 12.29	546.33 ± 49.22	374.22 ± 51.91	0.007^∗∗^
GSH-Px (U/g protein)	0.77 ± 0.01	0.42 ± 0.02	1.61 ± 0.05	0.79 ± 0.01	0.68 ± 0.02	0.58 ± 0.04	1.00 ± 0.04	0.96 ± 0.08	0.041^∗^
SOD (*μ*g/g protein)	30.28 ± 5.11	28.53 ± 7.04	30.33 ± 5.21	39.44 ± 7.11	44.19 ± 11.04	31.56 ± 6.22	25.91 ± 4.22	27.77 ± 8.07	0.071

^∗^*p* < 0.05; ^∗∗^*p* < 0.01. LPO: lipid peroxidation; CC: protein carbonyl content; GSH: reduced glutathione; catalase; GSH-Px: glutathione peroxidase; SOD: superoxide dismutase.

**Table 5:** Estimation of tissue homogenate lipid peroxidation (LPO), reduced glutathione (GSH), catalase, glutathione peroxidase (GSH-Px), and superoxide dismutase (SOD) in kidney for all groups.

	Control group	Toxin group	Lipid group	Lipid+toxin group	CHO group	CHO+toxin group	Protein group	Protein+toxin group	*p* value
LPO (nmol/g)	24.55 ± 4.08	60.44 ± 9.05	14.31 ± 3.03	18.40 ± 4.64	28.31 ± 3.04	48.27 ± 11.05	33.70 ± 6.09	40.28 ± 4.79	0.049^∗^
GSH (nmol/g)	4.80 ± 1.01	1.37 ± 0.61	7.48 ± 1.02	9.73 ± 0.71	8.11 ± 0.58	5.59 ± 2.60	3.21 ± 0.30	7.29 ± 0.41	0.024^∗^
Catalase (U/g)	81.02 ± 16.08	24.45 ± 6.21	28.30 ± 3.07	230.00 ± 27.11	124.81 ± 21.04	134.02 ± 32.11	217.40 ± 49.22	220.22 ± 31.44	0.007^∗∗^
GSH-Px (U/g protein)	0.66 ± 0.02	0.41 ± 0.03	0.98 ± 0.03	0.50 ± 0.01	0.45 ± 0.02	0.61 ± 0.04	0.81 ± 0.04	0.55 ± 0.10	0.062
SOD (*μ*g/g protein)	23.73 ± 5.05	20.72 ± 2.80	23.99 ± 4.44	20.70 ± 3.28	40.83 ± 8.01	33.20 ± 5.04	12.01 ± 2.09	14.82 ± 3.50	0.070

^∗^*p* < 0.05; ^∗∗^*p* < 0.01. LPO: lipid peroxidation; CC: protein carbonyl content; GSH: reduced glutathione; catalase; GSH-Px: glutathione peroxidase; SOD: superoxide dismutase.

**Table 6:** Lipid peroxidation (LPO), reduced glutathione (GSH), catalase, glutathione peroxidase (GSH-Px), and superoxide dismutase (SOD) in cardiac tissue for all groups.

	Control group	Toxin group	Lipid group	Lipid+toxin group	CHO group	CHO+toxin group	Protein group	Protein+toxin group	*p* value
LPO (nmol/g)	30.67 ± 4.61	41.76 ± 6.69	15.04 ± 2.70	18.84 ± 4.07	25.08 ± 5.80	23.71 ± 2.46	10.79 ± 2.17	22.05 ± 4.10	0.032^∗^
GSH (nmol/g)	1.42 ± 0.34	0.81 ± 0.05	2.81 ± 0.17	1.99 ± 0.41	1.57 ± 0.60	2.09 ± 0.28	8.59 ± 0.64	7.33 ± 1.02	0.009^∗∗^
Catalase (U/g)	70.11 ± 13.22	20.21 ± 3.07	238.04 ± 2.84	283.40 ± 25.06	183.30 ± 18.93	146.37 ± 41.15	314.60 ± 33.48	414.20 ± 38.05	0.004^∗∗^
GSH-Px (U/g protein)	0.45 ± 0.01	0.22 ± 0.02	0.88 ± 0.04	0.86 ± 0.01	0.44 ± 0.02	0.41 ± 0.04	0.31 ± 0.03	0.61 ± 0.14	0.051
SOD (*μ*g/g protein)	30.33 ± 4.02	21.09 ± 3.91	28.55 ± 3.22	26.06 ± 2.56	24.68 ± 2.04	30.12 ± 4.69	29.33 ± 7.22	21.39 ± 3.07	0.084

^∗^*p* < 0.05; ^∗∗^*p* < 0.01. LPO: lipid peroxidation; CC: protein carbonyl content; GSH: reduced glutathione; catalase; GSH-Px: glutathione peroxidase; SOD: superoxide dismutase.

## Data Availability

The datasets used and/or analyzed during the current study are available from the corresponding author upon request.
